# Microfluidic point-of-care testing for antimicrobial resistance: advances, strategies, and One Health perspectives

**DOI:** 10.1016/j.soh.2026.100160

**Published:** 2026-05-14

**Authors:** Chenxi Wang, Jun Feng, Chenjia Zhou, Leshan Xiu, Qinqin Hu, Hui Li, Xiaokui Guo, Xu Wang, Min Chen, Kun Yin

**Affiliations:** a School of Global Health, Chinese Center for Tropical Diseases Research, Shanghai Jiao Tong University School of Medicine, Shanghai, 200025, China; b Shanghai Municipal Center for Disease Control and Prevention, Shanghai, 200336, China; c School of Public Health, Shanghai Jiao Tong University School of Medicine, Shanghai, 200025, China; d School of Electrical, Computer and Biomedical Engineering, Southern Illinois University Carbondale, Carbondale, IL 62901, USA; e Department of Pathology, College of Basic Medical Sciences, Shanghai Jiao Tong University School of Medicine, Shanghai, 200025, China

**Keywords:** Antimicrobial resistance, Microfluidic system, Point-of-care testing, Antibiotic resistance gene, Antimicrobial susceptibility testing

## Abstract

Antimicrobial resistance (AMR) stands as a pressing global public health concern, particularly under One Health concept, requiring innovative approaches for effective treatment and control. In this context, point-of-care testing (POCT) emerges as a critical strategy to combat this challenge. Microfluidic systems have the potential to improve AMR management by enabling rapid, accurate, and cost-effective diagnostic strategies toward POCT for early detection and treatment guidance. This review first summarizes the current status of sample processing, manufacturing techniques, design and detection strategies in microfluidic systems. It then highlights the recent advances in microfluidic diagnostics for AMR, including microfluidic platforms for antibiotic resistance genes (ARGs) detection, and antimicrobial susceptibility testing (AST). Finally, the challenges and perspectives for the further development of microfluidic technology in POCT for AMR are discussed under the One Health concept.

## Graphical abstract

**Figure:**
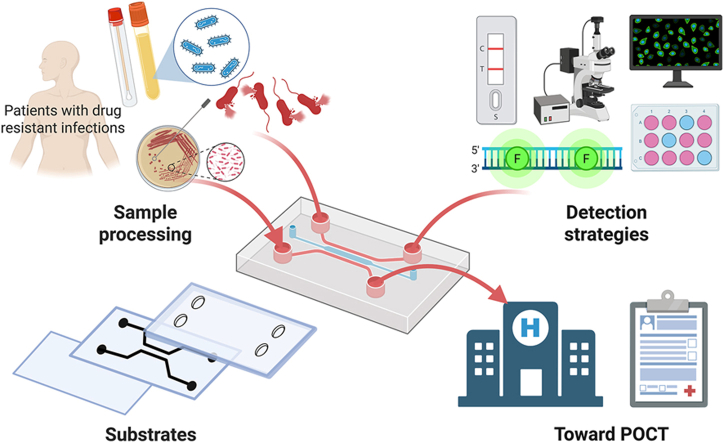

## 1 Introduction

Antimicrobial resistance (AMR) is a significant One Health concern driven by the selection and transmission of resistant organisms under antimicrobial pressure [1]. Without appropriate intervention, AMR could cause 10 million deaths worldwide annually by 2050, with a cumulative economic impact of approximately $100 trillion lost to global Gross Domestic Product (GDP) [2,3]. A major contributor to this global threat is the reliance on empirical antibiotic therapy caused by the lack of rapid and reliable diagnostic tools, which accelerates the selection and dissemination of resistant strains [[4], [5], [6], [7]]. Accurate antibiotic prescription and management can be hindered by the limitations of conventional diagnostic methods. For example, techniques such as antimicrobial susceptibility testing (AST), PCR, and whole genome sequencing (WGS) require either costly equipment, highly skilled personnel, long turnaround times, or a combination of these factors. For example, conventional techniques for AST vary widely in their requirements and performance. Broth microdilution, the gold-standard AST method, is time-consuming (typically 16–24 h) but requires minimal specialized equipment. In contrast, microscopy-based AST has been reported to provide rapid single-cell analysis (within hours) but demands skilled personnel and advanced instrumentation. Similarly, PCR can enable rapid detection of resistance genes (within 2–4 h), though it may miss phenotypic resistance mechanisms not linked to known genetic markers. WGS offers comprehensive insights but remains relatively costly and computationally intensive, although recent advancements in nanopore sequencing have reduced the turnaround time for pathogen identification and selected genomic analyses to as little as 8 h in some workflows [8]. These trade-offs highlight the need for adaptable, cost-effective alternatives in resource-limited settings. This severely limits their availability and practicality for clinical application, especially in areas with resource-limited settings [9,10]. AMR is not only a clinical challenge, but also a One Health issue shaped by the interconnected transmission of resistant bacteria and resistance genes among humans, animals, food systems, and the environment. In this broader context, microfluidic diagnostic technologies may support not only clinical testing, but also decentralized surveillance and rapid detection across diverse settings. Beyond human clinical diagnostics, the potential value of microfluidic AMR detection should be considered within a broader One Health surveillance framework. In principle, these miniaturized and integrated systems could support AMR monitoring in veterinary specimens, food matrices, and environmental samples, where rapid and decentralized testing remains limited. Although most reported platforms are still at the proof-of-concept stage and have been developed mainly for clinical use, their portability, low reagent consumption, and multiplexing potential make them promising candidates for future cross-sectoral AMR surveillance.

To address these challenges, there is a growing need to develop rapid, cost-effective, and user-friendly diagnostic methods that enable timely AMR detection and massive screening. The World Health Organization (WHO) has initiated a global report suggesting that by 2020, all clinicians should perform a rapid diagnostic test before prescribing antimicrobials [2]. Point-of-care testing (POCT) is a potential candidate that can significantly improve patient outcomes, enable appropriate treatment decisions, and support AMR surveillance [11]. An ideal POCT platform should be rapid, affordable, easy to operate, and capable of integrating sample preparation, amplification, and detection within a compact format. Nowadays, microfluidics has demonstrated the possibility of integrating sample preparation and target detection into a single miniature system, offering a promising opportunity for POCT or near-patient applications [[12], [13], [14], [15]]. Its small size and flexible design significantly reduce turnaround time, simplify the procedure, and enable multiplex detection [16,17]. The application of microfluidic systems in AMR diagnostics heralds a paradigm shift in combating this urgent global challenge.

Several studies have investigated the feasibility of employing microfluidics for rapid detection of AMR [12,18,19]. Nonetheless, it is shown that a majority of these platforms are still under development in laboratory settings, and not meeting the ASSURED (affordable, sensitive, specific, user-friendly, rapid, equipment-free, delivered) principles of POCT outlined by the WHO [20]. Therefore, there is still a gap in tracking the development of microfluidic AMR detection systems, identifying shortcomings, and presenting future perspectives through a comprehensive review. We anticipate that this article will be of great benefit to diagnostic developers who are seeking to establish a POCT approach for maintaining efficient detection and control of AMR. However, the practical implementation of these systems in point-of-care settings depends not only on analytical performance, but also on sample type, degree of workflow integration, instrument requirements, ease of operation, and compatibility with decentralized clinical or field use. This review provides a narrative and critical overview of representative microfluidic technologies relevant to AMR diagnostics. The discussion focuses on four major aspects: sample processing, substrate materials, detection strategies, and translational challenges toward practical implementation. In this article, POCT is discussed mainly as a translational direction and design goal for microfluidic AMR diagnostics. However, many currently reported platforms remain at the laboratory proof-of-concept stage and do not yet function as true point-of-care systems.

## 2 Sample processing in microfluidic systems

**Fig. 1:**
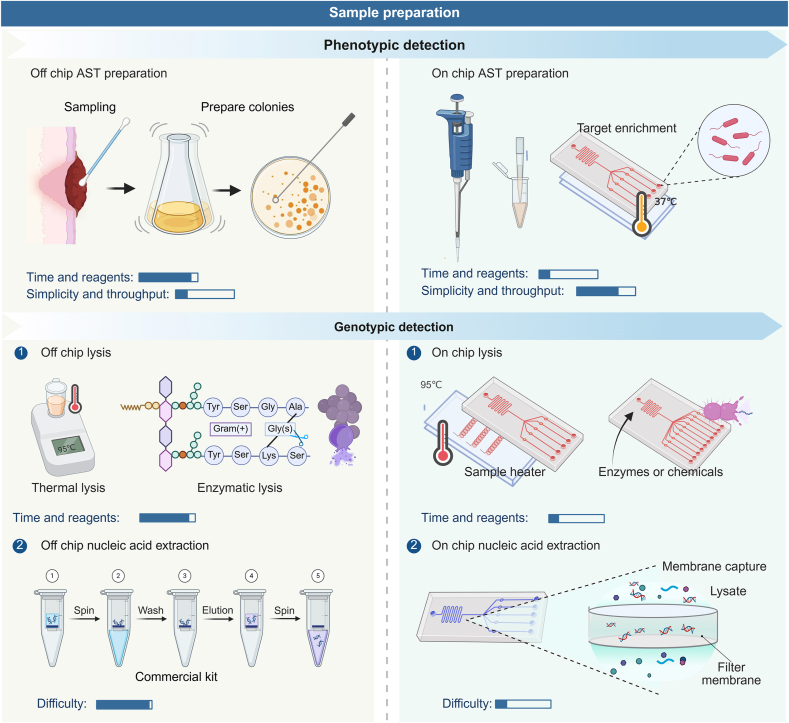
Sample preparation in microfluidic systems. Abbreviation: AST, antimicrobial susceptibility testing.

Effective sample preparation is critical for successful downstream AMR detection [21]. In general, sample preparation protocols are divided into two main categories depending on the purpose of the study: colony culture for phenotypic AST investigations [[22], [23], [24], [25], [26], [27], [28]], and cell lysis combined with nucleic acid extraction for genotypic analyses [29]. Notably, microfluidic systems for sample handling have exhibited remarkable advancements in terms of cost-effectiveness and ease of use for both categories (Fig. 1). For phenotypic studies, microfluidics enables high-throughput single-cell culture under controlled conditions, allowing precise monitoring of bacterial growth and antibiotic response at reduced reagent volumes [[30], [31], [32]]. In genotypic analysis, integrated microfluidic platforms combine rapid cell lysis, nucleic acid extraction, and on-chip amplification, streamlining the detection of resistance genes within a single device. These systems minimize cross-contamination risks, reduce processing time, and enhance portability for point-of-care applications. In addition to supporting genotypic assays, microfluidic devices can also isolate and concentrate pathogens from complex specimens for downstream phenotypic AST. Recent studies have demonstrated streamlined workflows for bacterial separation from clinical matrices such as urine, enabling more direct phenotypic susceptibility analysis with reduced manual preprocessing [33,34].

For phenotypic AST, the main technical advances in sample processing include rapid bacterial enrichment, on-chip confinement, and miniaturized cultivation under controlled conditions. Standard AST requires at least 24 h to grow bacterial cultures and another 18–24 h to measure antibiotic effectiveness, making the process slow and labor-intensive [35]. Microfluidic technology offers a transformative solution through on-chip target enrichment of bacteria within minutes, eliminating the necessity for bacterial colony isolation. This innovation significantly streamlines the testing procedure, achieving notable time savings [[30], [31], [32]]. The microfluidic nanogap capture principle demonstrated by Busche et al. [32] enables sample pre-concentration by allowing on-chip filtration and the subsequent proliferation of bacteria. This nanogap can capture a sufficient number of cells from a bacterial dilution containing 10^4^ cells/mL within 10 min, suggesting that the platform is suitable for the detection of uncultured and low-concentration samples. Chen et al. [31] employed the synergy of dielectrophoresis and electroosmotic flow forces for rapid enrichment and concentration, achieving a thousand-fold target enhancement from samples within 2 min and complete on-chip AST in about 2 h, with a limit of detection (LOD) of 3 colony forming unit (CFU)/mL. This is five orders of magnitude more sensitive than what can be achieved through the standard centrifugation-purification process. In addition, the compact design of microfluidic systems has led to a significant reduction in reagent consumption, particularly the large volumes of media and antibiotics used in conventional AST. A novel microfluidic platform was developed for rapid and automated AST [36], which requires only nanoliter reagents for AST of seven antibiotics and the accurate determination of minimum inhibitory concentration (MIC). Together, these studies show that microfluidic sample processing for phenotypic AST is moving toward faster enrichment, lower reagent consumption, and more integrated testing workflows.

For genotypic detection, microfluidics offers a promising solution for integrating and accelerating bacterial lysis and target enrichment, which can reduce the turnaround time and indirectly improve the detection capability. Clinical samples often contain complex matrices and low-abundance targets, which severely limit the performance of conventional molecular assays. Microfluidic platforms address these challenges by enabling precise fluid handling, rapid heat transfer, and efficient surface-based capture of nucleic acids. For example, Valiadi et al. [37] developed a microfluidic system for sample preparation. The protocol includes a 5 min incubation of the urine sample, a 3.5 min concentration of the sample from 1 mL to 6.4 μL, and a 5 min heat lysis, achieving complete sample preparation within only 15 min. This reduces the sample preparation time by one-third compared to commercially available nucleic acid extraction kits [38]. In addition, the integration of a target enrichment step into microfluidics significantly increases the assay sensitivity. For example, a microfluidic chip integrating a solid-phase extraction (SPE) chamber was established to concentrate DNA on acid-washed silica beads, achieving high sensitivity to 1 CFU/mL [39]. A nanoporous aluminum oxide membrane (AOM) filter was employed to improve DNA capture, enabling rapid enrichment of nucleic acids on microfluidic platforms and achieving a LOD of 10 copies/μL [40]. Consequently, microfluidic platforms provide time-saving and user-friendly alternatives to conventional AMR detection methods, making them more available for resource-limited settings [39]. Once sample handling and target preparation are integrated on chip, the choice of substrate becomes the next key determinant of device performance, as it influences fabrication strategy, fluid control, optical compatibility, and suitability for point-of-care deployment.

## 3 Substrates of microfluidic systems

**Fig. 2:**
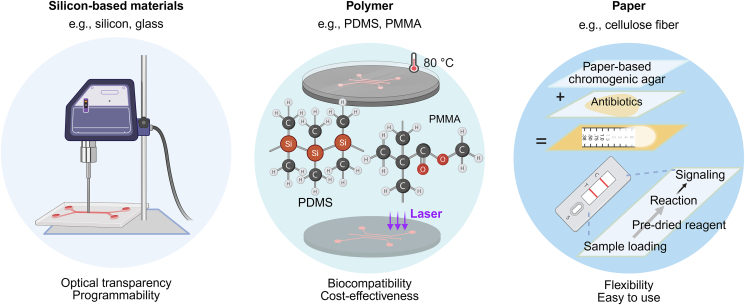
Microfluidic systems with different substrate materials. Abbreviations: PDMS, polydimethylsiloxane; PMMA, polymethyl methacrylate.

Nowadays, a series of microfluidic systems have been established using various substrates including silicon-based materials, polymers, and paper towards point-of-care detection of AMR [41] (Fig. 2; Table 1). Silicon-based microfluidic chips can offer satisfactory optical transparency when bonded to transparent polymers or glass and good compatibility with precise manufacturing processes, enabling real-time monitoring and accurate detection [42]. Polymers offer flexibility and cost-effectiveness making them suitable for disposable platforms [43]. The paper-based microfluidics owns portability and simplicity, meeting the needs of resource-limited settings [44]. The advancements in microfluidic systems substrates contribute to the development of microfluidic chips for the POCT of AMR. Recent studies have further expanded the fabrication landscape of microfluidic devices, with growing attention to advanced 3D fabrication and hybrid-material integration beyond conventional laboratory prototyping [45,46].

**Table 1:** Diversified substrates useful for different AMR detection purposes.

Substrate	Target	Sensor type	LOD	Time	Reference
Silicon	ARG	Fluorescence	10 CFU/mL	1 h	[47]
	ARB	Microscopy	NA	NA	[32]
Glass	ARG	Fluorescence	1 copy	15 min	[48]
		Fluorescence	10 copies	25 min	[49]
	ARB	Extracellular oxygen consumption	NA	∼6 h	[50]
		Fluorescence	NA	3–8 h	[51]
		Fluorescence	NA	18 h	[52]
		Microscopy	NA	3 h	[53]
		SERS	3 CFU/mL	2 h	[31]
PDMS	ARG	Colorimetry	NA	24 h	[54]
		Fluorescence	<10 CFU/mL	2 h	[39]
		Fluorescence	10 CFU/mL	1 h	[55]
	ARB	Colorimetry	NA	15 h	[56]
		Colorimetry	100 CFU/mL	24 h	[57]
		Fluorescence	30 fg/μL	2.5 h	[40]
		Fluorescence	NA	1 h	[58]
		Fluorescence	NA	NA	[59]
		Fluorescence	One cell	2–4 h	[60]
		Fluorescence	NA	1.5 h	[61]
		Fluorescence	NA	∼8 h	[62]
		Fluorescence	NA	∼5 h	[63]
		Fluorescence	10 CFU/mL	4–8 h	[64]
		Fluorescence	NA	2–4 h	[65]
		Fluorescence	NA	∼5 h	[66]
		Fluorescence	NA	∼1 h	[67]
		Fluorescence	NA	4–5 h	[68]
		Fluorescence	NA	8–9 h	[36]
		Microscopy	NA	3–4 h	[69]
		Microscopy	NA	1 h	[30]
		Microscopy	NA	5 h	[70]
		Microscopy	NA	3 h	[71]
		Microscopy	NA	3 h	[72]
		Microscopy	NA	33 min	[73]
		Microscopy	NA	30 min	[74]
		Microscopy	NA	2–3 h	[75]
		Microscopy	NA	2 h	[35]
		Microscopy	NA	3–4 h	[76]
		Microscopy	NA	1–3 h	[77]
		Microscopy	10 CFU/mL	∼2 h	[78]
		Optical density	NA	2–4 h	[79]
		Scattering	NA	NA	[80]
		SERS	10^3^ CFU/mL	2 h	[81]
		SERS	10^3^ CFU/mL	NA	[82]
		SERS	NA	5 h	[83]
PMMA	ARG	Colorimetry	10^4^ CFU/mL	3 h	[84]
		Fluorescence	1000 bacteria/mL	45 min	[37]
	ARB	Colorimetry	NA	24 h	[85]
		Colorimetry	NA	9 h	[86]
		Colorimetry/fluorescence	10^3^ CFU/mL	5 h	[87]
		Colorimetry	NA	4–9 h	[88]
		Fluorescence	10^4^ CFU/mL	30 min	[89]
		Fluorescence	NA	2 h	[90]
		Microscopy	NA	4 h	[91]
		SERS	10^3^ CFU/mL	NA	[92]
PC	ARG	Fluorescence	20–200 CFU/reaction	70 min	[93]
	ARB	Fluorescence	30 fg/mL	40 min	[94]
PP	ARB	Microscopy	NA	1–3 h	[15]
PTFE	ARB	Optical density	NA	1 h	[14]
VeroClear	ARB	Microscopy	5 × 10^3^ cells/mL	2 h	[95]
Paper	ARB	Colorimetry	NA	Overnight	[96]
		Colorimetry	5 × 10^3^ copies	1 h	[97]

Abbreviations: PDMS, polydimethylsiloxane; PMMA, polymethyl methacrylate; PC, polycarbonate; PTFE, poly tetra fluoroethylene, PP, polypropylene; LOD, limit of detection; ARB, antimicrobial resistant bacteria; ARG, antibiotic resistance gene; SERS, surface enhanced Raman spectroscopy; CFU, colony forming units; NA, not applicable.

As summarized in Table 1, polymer-based platforms, particularly polydimethylsiloxane (PDMS) and polymethyl methacrylate (PMMA), account for a large proportion of reported microfluidic systems for AMR detection, reflecting their advantages in fabrication flexibility and cost-effectiveness. In addition, most reported platforms are designed for phenotypic analysis of antimicrobial-resistant bacteria rather than solely for resistance gene detection, indicating the strong interest in rapid AST applications. Fluorescence- and microscopy-based readouts are the most commonly used strategies, whereas surface enhanced Raman spectroscopy (SERS), optical density, and other sensing modes remain less common but offer complementary advantages in specific settings. Although the reported assay times vary considerably, many studies have reduced detection or susceptibility testing to within a few hours, highlighting the potential of microfluidics to accelerate AMR diagnostics. These trends also suggest that substrate selection is closely linked to the intended application scenario, with polymer- and paper-based systems showing particular promise for low-cost and decentralized testing.

### 3.1 Silicon-based materials

Silicon exhibits both thermal and chemical stability, making it an attractive candidate for microfluidics fabrication that requires durability for heating and delicate operations. Due to its crystalline structure, silicon enables anisotropic etching with high resolution [42]. Silicon supports electrokinetic actuation (e.g., electrowetting, direct current/alternating current electroosmosis) due to its high thermal and electrical conductivity. The material’s rigidity allows for precise microfabrication of electrodes, enabling high-voltage applications such as droplet sorting or PCR thermal cycling. Pneumatic actuation is less common due to silicon’s non-deformability, though hybrid designs (e.g., silicon-PDMS bonding) can integrate external valves. Yao et al. [98] developed a silicon digital PCR chip with approximately 20,000 microchambers, which enabled seamless image acquisition and fusion with automated fluorescence imaging and realized precise quantitative analysis. However, the high cost of silicon material limits its attractiveness as a viable material for large-scale production. The limited transparency of silicon to visible and ultraviolet light impacts its usability for the mainstream fluorescence-based or microscopy imaging readout. Seeking alternatives to silicon, researchers redirected their focus towards glass [99].

Glass is an ideal material with optical transparency and low fluorescence background, enabling the incorporation of light-based detection into devices [99]. More importantly, when coated with indium tin oxide (ITO) glass, glass-based microfluidics enables electronic control and manipulation of fluid behavior within the channels, contributing to programmable functions. For example, Kalsi et al. [48,49] designed digital microfluidic systems comprising a thin film transistor, ITO glass, and an ionic barrier insulator layer formed from Al_2_O_3_, realizing complex fluid manipulation of ARGs detection by all-electronic control. Typically, the significant increase in pixel quantity for flexible droplet control would require a considerable number of signal lines, as well as complicated control circuitry. Digital microfluidics (DMF) can manipulate discrete droplets electrostatically using arrays of insulated electrodes [100]. By sequentially activating adjacent electrodes, individual droplets can be transported, merged, split, or mixed in a programmable manner on the chip surface. This mode of manipulation is particularly attractive for AMR diagnostics because it enables automated low-volume reactions, flexible assay design, and parallel processing with reduced reagent consumption. The “pixel quantity” refers to the number of independently addressable electrodes in the DMF array, which determines the resolution and flexibility of droplet control (e.g., splitting, merging, and transport). A higher pixel density enables more precise and complex fluidic operations. However, researchers addressed the scalability issues of digital microfluidic arrays by employing the active matrix technology in conjunction with a thin film transistor substrate. The scalability to perform high-throughput analysis on a microfluidic chip allows for the simultaneous processing of multiple samples, which can increase the testing capacity and efficiency for POCT.

### 3.2 Polymer

In recent years, polymers have been widely adopted in microfluidic applications. Among them, PDMS is one of the most widely used substrates with many desirable properties (e.g., low price, easy molding, low autofluorescence, and good biocompatibility) [101]. In addition, the air permeability of PDMS can be exploited for degassing-driven automatic sample loading, which reduces the reliance on bulky external pumps and may improve the feasibility of on-site AST. It is ideal for pneumatic and vacuum-driven actuation because of its elasticity, enabling integrated Quake valves for droplet generation or peristaltic pumping. The fabrication of functional PDMS microfluidic platforms involves several important steps: design, fabrication of the master mold, PDMS preparation and curing, substrate bonding, inlet/outlet ports creating, and final assembled testing. Soft lithography emerges as a prominent technique for fabricating PDMS microfluidic chips [102]. The technique encompasses a group of patterning methods that utilize replica molding, involving a few simple steps to create a PDMS mold [103]. Briefly, a rigid master is fabricated, typically using photolithography, and rapid prototyping methods such as injection molding, hot embossing, or laser printing can also be employed. Next, uncured PDMS is modeled on the master through methods such as casting [71] or spin coating [60]. Subsequently, the chip layer is cured at a high temperature and peeled off from the model for further processing or assembly [73].

Besides PDMS, PMMA is another popular polymer in microfluidics, which is characterized by high dimensional stability, low autofluorescence, and good optical properties [104,105]. Thermoplastics like PMMA are better suited for centrifugal microfluidics, as their rigidity withstands rotational forces. In certain cases, PMMA serves as a waveguide and substrate to facilitate visual microscopic observation [84]. PMMA-based microfluidics are usually fabricated by injection molding, and laser cutting to obtain intricate shapes and patterns [92]. Of these two techniques, laser cutting stands out for its ability to create highly precise and accurate designs, which are difficult to achieve with other methods [42]. To reduce the risk of thermal damage to the material during laser cutting, computer numerical control has also been used in PMMA fabrication recently [42]. The scalability and speed of computer numerical control machining allow for the production of microfluidic systems on a large scale with consistent quality, enabling complex designs tailored to specific applications in PMMA-based AMR detection devices [92].

Polycarbonate (PC) stands out as a promising material due to its exceptional thermal stability. With a high glass transition temperature ranging between 150 and 155 °C, PC is ideal for applications that require high temperatures [106]. To demonstrate this potential, Meng et al. [93] established a PC-based microfluidic system capable of distinguishing various staphylococcal species and predicting methicillin resistance by loop-mediated isothermal amplification (LAMP) reaction.

### 3.3 Paper

In recent years, the field of paper-based microfluidics has emerged as a promising avenue for POCT. Benefiting from the advantageous features of paper including its disposability and affordability, it can be easily transported, distributed and safely incinerated [107]. Paper’s combustion byproducts (primarily CO_2_ and water) are thus far less hazardous, aligning with waste disposal regulations and circular economy goals. In addition, the porous nature of paper enables automated fluidic handling, while the user-friendly visual result readout, usually via lateral flow strips, further enhances the appeal of paper-based microfluidic systems [108]. Lafleur et al. [97] developed a fully integrated platform featuring a paper-based signal readout for the first time, optimally suited for relevant real-world applications. The platform contains a paper network that is physically multiplexed to allow independent isothermal amplification of multiple targets. After amplification, solutions are moved to the lateral flow strip for the detection of *ldh1* and *mecA*. This design takes advantage of the capillary action of paper, circumventing the limitations of the external pump or power source. In addition to lateral flow devices, paper-based chromogenic agars are utilized for culture-based bacteria identification and AST [96]. He et al. [96] developed a microfluidic device that integrates paper-based chromogenic agar for bacterial culturing, boasting user-friendliness and adaptability across various settings, particularly those with limited resource. These studies underscore the promise of paper-based microfluidics for low-cost AMR testing, particularly in settings where simplicity and portability are important. Beyond their simple readout principle, colorimetric methods are particularly attractive for point-of-care AMR diagnostics because they often require minimal instrumentation and can be interpreted visually or with low-cost portable devices. These features make them especially suitable for decentralized testing, primary healthcare settings, and resource-limited environments where access to advanced optical systems may be restricted. Overall, the selection of substrate materials in microfluidic AMR diagnostics depends not only on their intrinsic physical properties, but also on the intended diagnostic application. Silicon and glass are generally more suitable for systems requiring precise fabrication, stable thermal performance, or advanced optical/electronic integration, whereas polymer-based materials are more attractive for low-cost fabrication, flexible design, and disposable device development. Paper-based platforms are especially promising for point-of-care and resource-limited settings because of their low cost, portability, and equipment-free fluid handling. Therefore, material selection should be guided by a balance among analytical requirements, manufacturability, cost, and practical suitability for decentralized testing.

## 4 Detection strategies

**Fig. 3:**
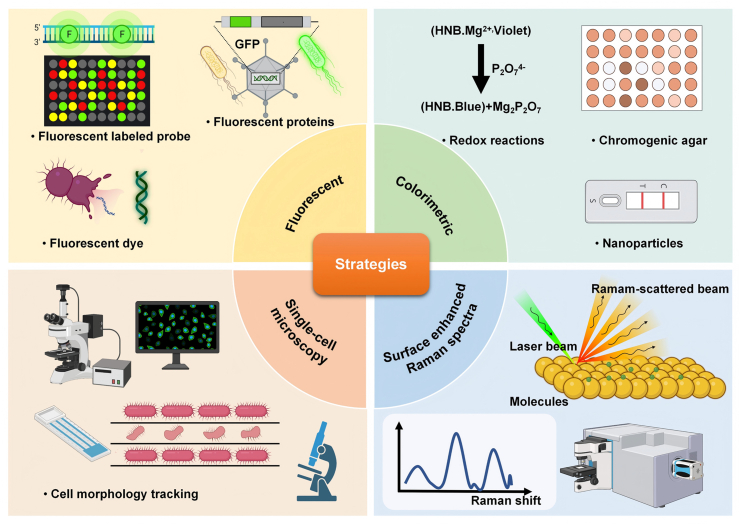
The mainstream readout forms in microfluidics. Abbreviations: GFP, green fluorescent protein; HNB, hydroxynaphthol blue.

As the final analytical step of the microfluidic workflow, the detection strategy determines how biological responses or resistance markers are translated into measurable signals. Its compatibility with chip design and operational requirements is therefore essential for building practical AMR diagnostic platforms. The following section highlights representative examples of how these readout strategies have been applied in AMR diagnostics, including resistance gene detection and phenotypic AST. Currently, fluorescence, colorimetry, Raman spectroscopy, and microscopic observation are the mainstream detection strategies in microfluidic AMR detection platforms [[109], [110], [111], [112]] (Fig. 3). A range of microfluidic systems facilitate both qualitative and quantitative investigations, yielding visual signals that can be conveniently interpreted by the naked eye or portable electronic devices like smartphones [[113], [114], [115]]. In addition, the development of Raman scattering and single-cell imaging techniques that are available for *in situ* analysis provides promising solutions for AMR identification and surveillance [116,117]. Here, we compile an overview of the distinctive attributes and pertinent research studies associated with diverse readout methods, empowering informed decision-making when selecting the optimal readout approach.

### 4.1 Fluorescence-based detection

In microfluidics, fluorescent signals are commonly used as readouts to detect and quantify specific AMR biomarkers. A promising approach involves using non-toxic dyes to selectively stain damaged cells [118]. Kalashnikov et al. [58] assessed the mortality rate of bacterial cells in the presence and absence of antibiotic stress as a means of distinguishing between susceptible and resistant *Staphylococci* using this method. This approach uses the direct addition of fluorescent dye to the cell culture medium, enabling the detection of damaged cells effectively without additional sample preparation steps. In an alternative fluorescence-based detection strategy, AMR pathogens are labeled with fluorescent proteins and the changes in fluorescence intensity indicate variations in cell numbers following antibacterial treatments [119]. For example, Mohan et al. [60] labeled *Escherichia coli* with green fluorescent protein, and detected fluorescence intensity over time, obtaining susceptibility results within 2–4 h. The microfluidic system efficiently evaluates the impact of four antibiotics and their combinations, elucidating the interplay between antibiotic effectiveness and combination therapy [60]. These findings highlight the potential benefits of identifying pathogen-specific antibiotic profiles with microfluidics for providing more precise antibiotic guidance in clinical treatment.

**Fig. 4:**
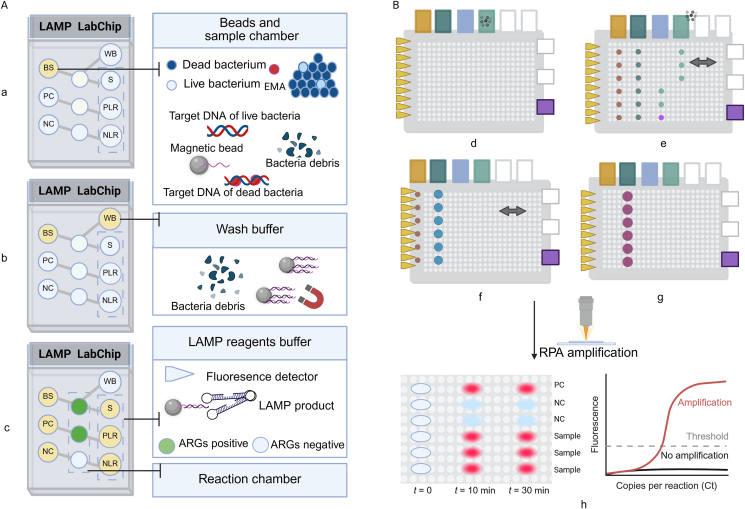
Fluorescence-based microfluidic readout system. A: The workflow of the integrated microfluidic system includes sample preparation and target DNA isolation (a), followed by a washing step (b), and conducting LAMP and collecting fluorescent signal (c) [47]. B: The DNA amplification workflow on the DMF platform. (d) Beads are moved through the oil in the DMF device onto the reservoir pad using an elution buffer. (e–g) Droplets containing reagents, controls, and samples are dispensed and mixed with RPA reagents. (h) Fluorescent signal collection [89]. Abbreviations: LAMP, loop-mediated isothermal amplification; BS, bead storage; PC, positive control; NC, negative control; WB, wash buffer; S, sample; PLR, positive LAMP reagent; NLR, negative LAMP reagent; EMA, ethidium monoazide; ARG, antibiotic resistance gene; DMF, digital microfluidics; RPA, recombinase polymerase amplification.

In addition, fluorescent signals can be generated based on the complementary hybridization reaction between ARGs and fluorescent probes or intercalating dyes, enabling the screening of resistance genes to monitor AMR [40,120]. For example, Chang et al. [47] used LAMP to detect *vanA* genes from living bacteria on a microfluidic chip. The fluorescent signal increases with the number of amplification cycles, enabling real-time monitoring of the amplification process by continuously measuring fluorescent signal (Fig. 4A). Kalsi et al. [89] proposed a recombinase polymerase amplification (RPA) assisted digital microfluidic molecular assay for the rapid detection of *bla*_CTX-M-15_, conferring antibiotic resistant *Klebsiella pneumoniae*. This platform can directly identify ARGs in human urine within 30 min, achieving a LOD of 10^4^ CFU/mL (Fig. 4B). In addition to membrane integrity dyes or fluorescently labeled bacteria, fluorescence-based phenotypic AST can also be achieved through enzyme-responsive substrates. Recent studies have shown that pathogen-associated or inducible enzymatic activities can trigger the hydrolysis of fluorogenic substrates after short antibiotic exposure, enabling rapid susceptibility determination and, in some cases, simultaneous bacterial identification [121,122].

### 4.2 Colorimetric-based detection

**Fig. 5:**
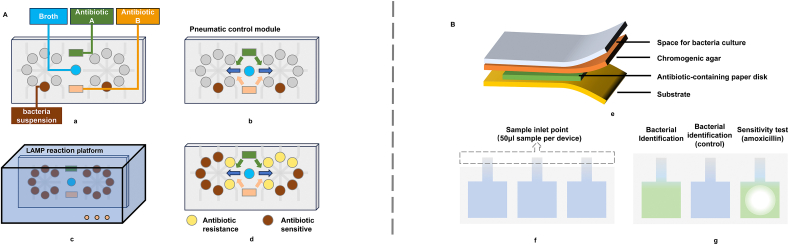
Colorimetric-based readout microfluidic systems. A: Illustrative diagram of colorimetric-based AST using antibiotic combinations [85]. (a) Loading of samples, pH-sensitive colorimetric broth, and two antibiotics. (b) Automated on-chip dispensing of bacteria, dilution and mixing of broth and antibiotics. This is achieved by varying pump durations, with longer durations resulting in higher reagent loads. The entire process is controlled by a custom pneumatic control module connected to the chip. (c) After incubating bacteria for 16–24 h, the AST results are interpreted visually. (d) A pH-triggered color shift from red to yellow if bacterial growth continues, or the maintenance of the red color if antibiotics have halted microbial growth. B: Schematic illustration of the three-layer paper device for bacteria identification and antibiotic-resistance testing. (e) Graphical representation illustrating the three-layer paper device. (f–g) The outcomes from combined paper-based devices: prior to testing with *Escherichia coli* pathogen (f), and after conducting antibiotic susceptibility tests using amoxicillin (g) [96]. Abbreviation: AST, antimicrobial susceptibility testing.

Due to their simplicity, speed, and cost-effectiveness, colorimetric detection has also been commonly utilized in microfluidic diagnosis of AMR [85,123,124]. Colorimetric assays can be completed with simple equipment, where color changes can be observed either by the naked eye or optical readers, making them well-suited for POCT. A notable colorimetric microfluidic platform is presented by Jin et al. [84], which uses hydroxynaphthol blue (HNB) to visualize the amplification of ARGs in LAMP reactions. The HNB yields a purple color initially and shifts to sky blue with reaction progresses. The integrated plastic chip and closed-tube detection platform enable the simultaneous identification of six ARGs in less than 2 h, achieving a LOD of 10^3^ CFU/mL. Colorimetric detection is now widely used in phenotypic AMR studies due to its ability to rapidly and sensitively detect changes in cell growth or metabolic activity. In Lee groups’ study [85] (Fig. 5A), the reagents, including bacterial suspensions, pH-dependent colorimetric broth, and antibiotics are first loaded into the chambers. The subsequent interaction between liquid chambers and diluted antibiotics of varying concentrations led to changes in medium color, allowing the determination of MIC values. In addition, the introduction of chromogenic agar provides a straightforward approach for the identification of infectious pathogens during AST. He et al. [96] proposed an innovative strategy by incorporating a chromogenic medium into their experimental design, which selectively facilitated the growth of *E. coli* (Fig. 5B). In their microfluidic systems, five antibiotics commonly used to treat urinary tract infections were tested simultaneously, resulting in the development of the first paper-based device capable of simultaneous bacterial culturing, species identification, and multiplexed AST.

### 4.3 Surface plasmon resonance-based detection

**Fig. 6:**
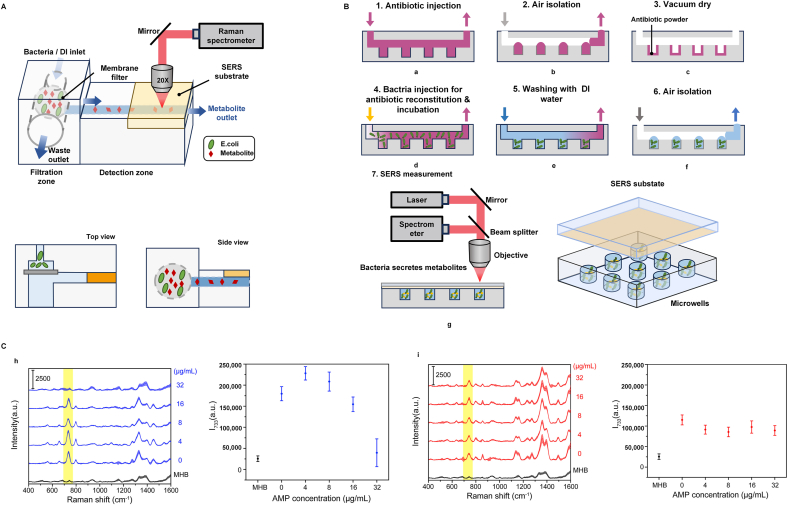
SERS-based readout microfluidic systems. A: Schematic illustration of a membrane filtration-assisted SERS platform (MF-SERS) for rapid AST, in which bacteria are enriched and analyzed through metabolite-associated Raman signals [92]. B: A microfluidic device operated by the automated microfluidic control system for SERS-based AST. (a–c) Antibiotic preloading involves antibiotic injection, followed by isolation and drying. (d) Bacteria injection for antibiotic reconstitution and incubation. (e–f) Deionized water washing and air isolation. (g) Attachment of SERS substrate for simultaneous SERS measurement. C: AST results of AMP-susceptible and AMP-resistant *Escherichia coli* treated with AMP for 3 h in the automated microfluidic control system: SERS spectra of the susceptible (h) and the resistant (h) strain treated with AMP [82] (reprinted from Biosensors and Bioelectronics, Vol. 191, C.C. Liao et al., A microfluidic microwell device operated by the automated microfluidic control system for surface-enhanced Raman scattering-based antimicrobial susceptibility testing, Pages 113,483, Copyright (2021), with permission from Elsevier). Abbreviations: AST, antimicrobial susceptibility testing; DI, deionized; SERS, surface enhanced Raman spectroscopy; AMP, antimicrobial peptide.

Raman spectroscopy is a label-free and non-invasive analytical technique that can provide characteristic fingerprints of molecules based on molecular vibrations and rotational information through the Raman scattering effect [125]. Compared to fluorescence and colorimetric methods, it does not require fluorescent labels or color indicators, reducing the use of chemical reagents and simplifying the experimental process [126,127]. Several studies have utilized SERS for AMR detection. For example, Chang et al. [92] developed a rapid AST platform with membrane filtration and SERS-active substrate (MF-SERS) by analyzing the SERS spectra of secreted metabolites (Fig. 6A). This proposed approach achieves a LOD of 10^3^ CFU/mL, which surpasses the sensitivity of traditional centrifugation-purification procedures by four orders of magnitude [92]. Despite the high detection performance of MF-SERS, the clinical utility is limited by the numerous off-chip sample preparation processes. Therefore, Liao et al. [82] developed an automated microfluidic system characterized by a control system to perform all AST steps (Fig. 6B). As the manual handling of reagents is eliminated, the system further reduces the risk of contamination. This high-throughput AST approach offers a sample-to-result time of 3.5 h, holding promise for efficient implementation. Lin et al. [83] presented an integrated chip for multiplex AST detection. The microfluidic system employs a Y-shaped main channel with 64 side channels to generate antibiotic concentration gradients and provide isolated microenvironments. The 792-microwell arrays under the side channel encapsulate bacteria in distinct spaces, enabling multiparallel SERS analysis (Fig. 6C). It is shown that integrating high-sensitivity spectroscopic detection with precise sample manipulation and parallel processing capabilities of microfluidics offers potential relevance for point-of-care diagnosis of AMR.

### 4.4 Microscopy imaging-based detection

**Fig. 7:**
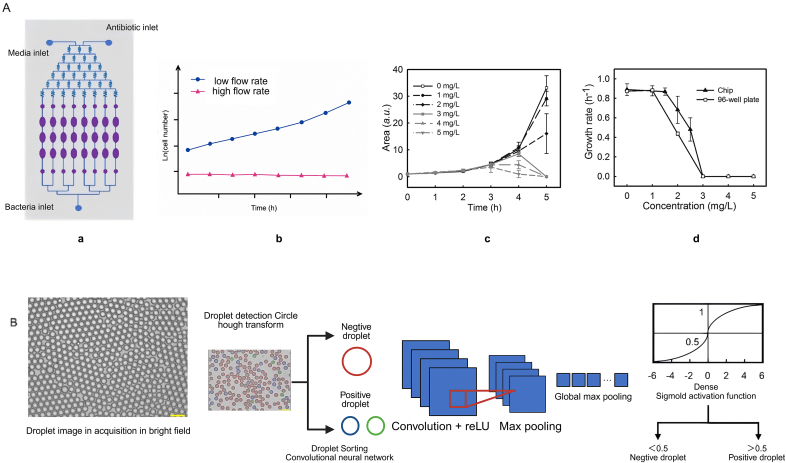
Microscopy imaging-based microfluidic systems. A: Schematic illustrations of a multiplexed microfluidic system [70]. (a) Microchannel structure featuring a Christmas tree design to create a concentration gradient. (b) Cell cultures at varying flow rates within the chip. (c) Variations in cell numbers were observed at different drug concentrations. (d) Variations in morphology lengths of *Escherichia coli* were observed at different drug concentrations. B: Workflow for positive and negative droplet classification and convolutional neural network structure used for fast and automated bacterial detection inside droplets (droplet images adapted from X. Zhou et al., WSCNet: biomedical image recognition for cell encapsulated microfluidic droplets, *Biosensors* 2023) 821, https://doi.org/10.3390/bios13080821, licensed under CC BY 4.0.) [134]. Abbreviation: reLU, rectified linear unit.

Molecular imaging and real-time monitoring of cells in confined spaces on microfluidics are among the most useful readout strategies. This technique enables the observation of dynamic changes in individual cells or populations, significantly reducing the time required for microbial growth quantification due to its ability to observe cells on a smaller scale [118,128,129]. Traditional microfluidic platforms that rely on microscopy typically perform agar dilution or broth dilution methods on microfluidic chips to observe bacterial growth for AST [27,130]. For example, a microfluidic AST device was developed by Samuel et al. [71], which is based on broth dilutions to generate gradient concentrations and perform cell culture in eight 30-nL chambers. The device can automatically perform AST through bacterial distribution, broth delivery, and antibiotic dilution. Recently, with the advancement of single-cell imaging technology, tracking the growth of individual cells has become particularly appealing for performing rapid and non-destructive AST without the need for culturing. Sun et al. [15] used microscopic imaging to monitor the number and morphology of bacterial cells to determine MIC values, achieving AST results within 1–3 h, which provides a practical solution for *in situ*, rapid, and label-free AST (Fig. 7A). In addition, deep learning for automated microscopic image analysis emerged as a more reliable tool for AST analysis [[131], [132], [133]]. Riti et al. [132] developed DropDeepL AST, which provides colistin susceptibility characteristics of clinical strains within 2 h and allows direct analysis of bacteria in urine samples at concentrations above the infection threshold. This method uses bright field imaging to detect the presence and growth of bacteria in droplets and automated image analysis is performed using deep learning algorithms. Further training of the relevant models will improve detection sensitivity and expand the range of detectable Gram-negative bacteria, facilitating timely prescription of targeted treatments for multidrug-resistant bacterial infections (Fig. 7B).

Overall, the suitability of different readout strategies for point-of-care AMR diagnostics depends on the balance between analytical performance and operational simplicity. Fluorescence-based methods provide high sensitivity and good compatibility with nucleic acid amplification, but usually require dedicated optical components. Colorimetric methods are more straightforward and inexpensive, making them particularly attractive for decentralized or resource-limited settings, although their quantitative resolution may be lower. Raman-based approaches offer label-free and information-rich detection, but their reliance on specialized instrumentation currently limits widespread point-of-care use. Microscopy-based methods are especially useful for rapid phenotypic AST through direct visualization of bacterial growth or morphology, yet their broader implementation depends on further progress in portable imaging and automated image analysis.

## 5 Challenges and perspectives

From a practical point-of-care perspective, the most promising applications at present are those involving relatively simple or clinically accessible sample matrices, such as urine and other low-complexity fluids, where sample pretreatment requirements are limited. By contrast, implementation in more complex specimens still faces major barriers, including pathogen isolation, target enrichment, matrix interference, and dependence on external instrumentation. Therefore, the translation of microfluidic AMR platforms into true point-of-care tools requires not only miniaturized chip design, but also simplified sample-to-answer workflows, portable readout systems, minimal hands-on steps, and robust performance under non-laboratory conditions. In recent years, the rapid development of microfluidic techniques has shown promising potential for the rapid and accurate AMR detection in various scenarios, ranging from the identification of clinical AMR pathogens to ARGs screening. Despite their popularity in recent years, these systems still struggle to meet the ASSURED criteria at the POCT [135]. Therefore, concerted efforts to refine and improve the POCT of AMR are imperative. The development of microfluidic-based AMR detection systems faces multifaceted challenges spanning technical, clinical, and regulatory domains. Technically, the intrinsic complexity of clinical samples demands sophisticated sample processing while maintaining device portability. Clinically, there exists an urgent need to bridge the gap between laboratory-performance and point-of-care robustness. From a regulatory perspective, the lack of standardized evaluation protocols hinders widespread adoption. Addressing these challenges could include developing more cost-effective and robust sensors, improving the completeness and accuracy of detection capabilities, and increasing the availability and accessibility of these devices to a wider range of healthcare providers and resource-limited settings.

### 5.1 Integrated sample pretreatment

Recent studies have demonstrated that microfluidics is capable of integrating sample pretreatment, facilitating the rapid detection of low-abundance AMR pathogens in clinical samples [31,32]. However, the development of such integrated platforms remains limited and faces two main challenges. Firstly, clinical samples are complex, requiring a series of steps to remove interfering substances and extract targets. Transferring steps to remove interfering substances from the microfluidic chip requires complex design or additional fluidic systems, making the development process more challenging [136]. Secondly, to detect low concentration pathogens in clinical samples, pre-enrichment steps such as membrane filtration and magnetic bead capture need to be introduced, which requires high precision in design and fabrication [10]. Overcoming these challenges requires the development of efficient target capture methods and advancements in high-precision chip fabrication techniques [28]. The integration of sampling onto microfluidic chips offers potential relevance for the development of rapid and portable point-of-care diagnostics. Addressing the existing challenges to improve the reliability and usability of microfluidics will be critical for realizing this potential and making these technologies available for widespread AMR detection applications.

### 5.2 Portable signal collection

Some current diagnostic methodologies are hampered by their dependence on specialized equipment including microscopes and Raman devices, particularly in resource-limited settings. Due to the lack of portability, these methodologies can only be used in laboratories, limiting the practical application of microfluidic methods in the field. Additionally, the high costs of required equipment underscore the financial limitations. Hence, suitable devices need to be developed for accessible alternatives. Zhang et al. [137] reported a portable Raman spectrometer for label-free and on-site detection of acquired AMR in *Salmonella typhimurium* in intact cells. The results indicate the potential application of Raman spectrum-based AMR detection in resource-limited settings. In recent years, the utilization of smartphones for signal observation has been widely employed in colorimetric and fluorescence-based assays [138], offering significant potential for the detection of various AMR markers in bacterial samples. The application of these devices can simplify the diagnostic process for healthcare workers with minimal training and make it more accessible in remote areas. Continued research and innovation are critical to overcoming AMR challenges and ensuring patients receive timely and appropriate treatment.

### 5.3 Multiplex detection

To facilitate informed decisions regarding the judicious use of antibiotics in clinical settings, microfluidic systems should improve the multiplexing capability for simultaneously screening different AMR biomarkers [68,76]. Dividing the chip into several independent chambers for different reactions is the most common strategy to realize multiplexed assays [[139], [140], [141]]. In these designs, different chambers can be preloaded with distinct antibiotics, concentrations, or target-specific reaction mixtures, allowing multiple testing conditions to be analyzed in parallel on a single chip. Although enabling simultaneous detection of multiple targets, these designs are limited by the number of reaction chambers and do not meet the requirements for large-scale assays. In this case, droplet microfluidics has emerged as a promising solution to perform multiple reactions by generating and manipulating sample and reagent droplets within the microfluidic system. The SCALe-AST platform utilizes picodroplet microfluidics to simultaneously evaluate 32 antibiotic conditions, employing physical barriers between droplet clusters to maintain <2% inter-group crosstalk. Through high-throughput statistical analysis of ∼10,000 droplets per antibiotic concentration, the system achieves single-bacterium resolution for accurate growth inhibition measurements [61]. This droplet-based design enables each droplet cluster to function as an isolated microreactor, thereby greatly increasing assay throughput while reducing reagent consumption and minimizing cross-contamination between testing conditions. By rapidly providing high-thought results, these innovative microfluidic approaches can serve as effective, time-saving, and accurate alternatives to conventional AST platforms commonly used in clinical diagnostics, ultimately benefiting patient care and treatment decisions. Recent advances have further expanded the role of droplet microfluidics in AST beyond simple reaction compartmentalization. By isolating individual bacteria or small populations into discrete droplets, these systems enable high-throughput screening of multiple antibiotic concentrations and conditions with reduced cross-talk and reagent consumption. More recent studies have combined droplet-based culturing with automated optical readout and data analysis, further improving assay throughput and supporting multiplex phenotypic AST at the single-cell level [132].

### 5.4 Process automation

Recent advancements in microfluidic automation are transforming AMR detection through integrated sample-to-answer systems that combine sample preparation, amplification, and detection in single cartridges. While current platforms like centrifugal LabDisk® systems achieve <5% coefficient of variation in gene quantification [142], key limitations persist including short reagent shelf-lives (<6 months at ambient temperatures), limited multiplex capacity (<10 targets/run), and elevated false-negative rates with viscous samples. Emerging solutions employ artificial intelligence (AI)-driven quality control [143], and self-powered platform (<15 min hands-on time) [144] could transform the field. Realizing these advances will require closer collaboration between microfluidic engineers, clinical microbiologists, and data scientists to balance technical feasibility with diagnostic needs.

Although numerous microfluidic systems have been reported for AMR detection, only a limited number have been validated with authentic clinical specimens under realistic use conditions. This limitation is particularly evident in the context of POCT. Many reported platforms are still based on cultured bacteria or simplified sample models rather than complex clinical specimens. In practice, true point-of-care AMR diagnostics remain challenging because pathogen isolation, target enrichment, and interference removal are often still required. At present, more realistic near-term applications may be limited to relatively accessible matrices such as urine, certain normally sterile fluids, and selected blood-based workflows. Therefore, broader clinical validation, standardized performance assessment, manufacturing reproducibility, and regulatory alignment will be essential before these systems can move from promising laboratory prototypes to practical diagnostic tools. Extending these platforms beyond human clinical diagnostics to broader One Health applications will be even more challenging, especially when considering heterogeneous non-clinical matrices such as animal fecal specimens, milk, meat products, wastewater, and surface water. Compared with relatively accessible clinical samples, these matrices often contain more complex background microorganisms, suspended solids, and amplification inhibitors, placing greater demands on sample pretreatment and assay robustness. Therefore, if microfluidic AMR systems are to contribute to integrated surveillance across veterinary medicine, food safety, and environmental monitoring, future development will require more effective pathogen enrichment, inhibitor removal, matrix-compatible extraction strategies, and validation in authentic non-clinical samples. Although such applications remain largely preliminary, they represent an important direction for extending microfluidic technologies from diagnostic tools to broader One Health AMR surveillance.

## 6 Conclusion

The development of cost-effective, fast and user-friendly microfluidic technologies for POCT of AMR under One Health is necessary to complement complex conventional methods. Recent advances in microfluidic design and the cutting-edge detection strategies have led to the development of microfluidic AMR detection and provide considerable promise for the POCT in resource limited settings. In this review, we systematically summarized the recent developments in microfluidic AMR detection and future perspectives of POCT application, which will contribute to overcoming existing limitations and promote innovation. As technological and process innovations continue to overcome existing limitations, microfluidic-based AMR POCT has the potential to transform how we use antimicrobial agents in humans, ultimately reducing unnecessary use, alleviating antibiotic resistance, and extending the lifespan of existing antibiotics. Future development of microfluidic technologies for AMR detection will likely focus on more fully integrated sample-to-answer systems, portable and low-cost signal readout, improved multiplexing capability, and greater automation of analysis workflows. In addition, advances in AI-assisted data interpretation, scalable manufacturing, and clinical validation will be important for translating promising laboratory prototypes into robust point-of-care tools. Continued progress in these directions may accelerate the deployment of microfluidic platforms in decentralized and resource-limited settings [10,143]. Further research and development in this area will be crucial for realizing the full potential of microfluidic-based POCT for AMR detection and management, particularly in the human-animal-environmental interface.

## CRediT authorship contribution statement

**Chenxi Wang:** Writing – original draft, Methodology. **Jun Feng:** Resources. **Chenjia Zhou:** Writing – original draft, Methodology. **Leshan Xiu:** Writing – original draft, Supervision, Methodology. **Qinqin Hu:** Methodology. **Hui Li:** Investigation. **Xiaokui Guo:** Resources, Methodology. **Xu Wang:** Methodology. **Min Chen:** Investigation. **Kun Yin:** Supervision, Investigation, Conceptualization.

## Funding

This work was sponsored by the the 10.13039/501100012166National Key R&D Program of China (2025YFC3409100), the Science and Technology Innovation Action Plan of Shanghai (grant number 24J22800900), the Interdisciplinary Program of Shanghai Jiao Tong University (project No. YG2024ZD02), Shanghai Pujiang Program (22PJD035), and the China Medical Board (23-526).

## Declaration of competing interest

The authors declare that they have no known competing financial interests or personal relationships that could have appeared to influence the work reported in this paper.
